# Evaluation of formal training in clinical ultrasonography and its utility in identifying treatable causes of PEA

**DOI:** 10.1186/2036-7902-4-S1-A21

**Published:** 2012-12-18

**Authors:** Ramon Nogué Bou, J Fabregat, R Vilella, A Encinas, T Villen, R Campos

**Affiliations:** 1 Hospital Montserrat, University of Lleida, Alcalde Sol, Nº 6-3, Lleida, Spain

## Background

Ultrasonography performed by the clinical physician should be a diagnostic tool, associated with anamnesis and physical examination of the patient, in order to increase security in the diagnoses, procedures, initial treatments and the following monitorization.

The medical areas that benefit most from this concept are those in which the time factor is important.

The present study examines aspects of standardized training clinical ultrasonography performed at the Faculty of Medicine, University of Lleida, in order to know the characteristics of the students, know their capacity to identify serious or fatal heart disease and the suitability of the view used in the videos (subcostal), all of them for the course and for clinical application.

## Material and methods

Standardized training in clinical ultrasonography, with a duration of 25 hours, for physicians without experience in ultrasonography and who attend patients with acute, urgent and critical conditions. The teaching methodology consists in a brief theoretical presentations followed by practical sessions in small groups (4-5 students) about ultrasonography, different benefits, monitor expert. They used live models and simulators. One hour of theory and two of practice are dedicated to echocardiography.

The course is assessed with a standardized practical and theoretical examination. Before an after de course all students respond a survey related to their previous knowledge, use and availability of this technique. A part of the evaluation consists of four videos of echocardiography in a subcostal view, 3 of them with life-threatening disease and a normal one, for 10 seconds. They had no available prior information of the case and only the image must be interpreted by the student and suggest a diagnosis.

Data was analyzed using SPSS v.17.

## Results

A total of 289 students attended to this course. 4 has been removed from or study because didn’t answered the exam or the survey, leaving a sample of 285 students. In the survey 95.5% rated as high the need for ultrasound in emergency services. There is a significant relationship (p <0.05) between the availability of radiologist in the hospital and the need to use ultrasound in the ED. 86.7% achieved optimal viewing at subcostal point. The 80.7% of students committed one or none error in the questions videos about heart disease. The following tables summarizes the number of students which correctly respond each video an in the response in that students who didn’t answered correctly. Tables 1 and 2.

**Table 1:** 

% of correct answers by video			
	Correct	Wrong	Total

Severe hypocontractility	208 (75.1%)	69 (24.9%)	277
Pericardial effusion	262 (93.9%)	17 (6.1%)	279
Without pathology	197 (72.7%)	74 (27.3%)	271
Right ventricle dilation	262 (93.2%)	19 (6.8%)	281

**Table 2:** 

Breakdown of the confusions in the wrong answered questions						
	Severe hypocontractility	Pericardial effusion	Without patology	Right ventricle dilation	Other answers	Total

Severe hypocontractility	-	7 (10.1%)	38 (55.1%)	14 (20.3%)	10 (14.5%)	69
Pericardial effusion	4 (23.5%)	-	9 (52.9%)	4 (23.5%)	0 (0.0%)	17
Without pathology	16 (21.6%)	16 (21.6%)	-	23 (31.1%)	19 (25/7%)	74
Right ventricle dilation	13 (68.4%)	1 (5.3%)	1 (5.3%)	1	4 (21.1%)	19

## Discussion and conclusions

95% of physicians who work in ED found useful the knowledge and use of ultrasonography. Those who work in hospitals considered more basic found the knowledge of this technique more useful. Most of the students achieved a correct visualization from the subcostal point, which correlates with other studies and publications in the bibliography. Would therefore be recommend as the initial view in a situation of AESP. The videos with a major percentage of errors are the severe hypocontractiblity and the healthy hearth, in contrast a more than 90% of students identified the pericardial effusion and the dilatation of right ventricle, which are potentially treatable diseases in CPR contexts. These results support the thesis that a short course can help to identify potentially treatable pathology in a PEA context.

